# Bridging the gap in aging systems: health literacy and community health worker workforce innovation to address health-related social needs

**DOI:** 10.3389/fpubh.2026.1952147

**Published:** 2026-09-02

**Authors:** Teresa Wagner, Sarah Murphy, Saphina Achi

**Affiliations:** 1 Department of Public Health, Texas College of Osteopathic Medicine, UNT Health Fort WorthFort Worth, TX, United States; 2 Center for Older Adults, Texas College of Osteopathic Medicine, UNT Health Fort WorthFort Worth, TX, United States

**Keywords:** CHWs, community health workers, health equity, health literacy, HRSN, older adults, workforce training

## Abstract

**Introduction:**

Older adults experience complex health and social challenges, including chronic disease, polypharmacy, caregiving demands, social isolation, and unmet health-related social needs (HRSN). Health literacy is a cross-cutting factor influencing the ability to find, understand, communicate about, and navigate health and social services. Community health workers (CHWs) are well positioned to address these intersections, yet integrated workforce training remains limited.

**Methods:**

This manuscript describes a health literacy-centered CHW and frontline workforce training model emphasizing dementia and depression screening, health literacy-informed communication, referral, and resource navigation. Developed collaboratively by the Health Literacy Lab, Community Health Worker Training Center, and Center for Older Adults at UNT Health Fort Worth, the model incorporated community-engaged curriculum development, interdisciplinary education, and cross-sector partnerships. A descriptive post-training program evaluation assessed knowledge, perceived skills, and intended application. Of 270 participants completing training, 201 completed the post-training assessment (74.4% response rate).

**Results:**

Mean post-training knowledge was 84.9% (SD = 9.23). Participants demonstrated strong knowledge of dementia, depression, health literacy, culturally responsive communication, and referral practices. Participants reported confidence applying health literacy-informed communication and screening strategies, and 167 (83.1%) intended to implement strategies addressed during training. Pre-training knowledge, observed practice change, HRSN resolution, and patient outcomes were not assessed.

**Discussion:**

Findings provide preliminary evidence supporting a health literacy-centered workforce development approach. The model positions health literacy as a cross-cutting competency relevant to HRSN screening, referral, and navigation and provides a framework for strengthening CHW capacity across healthcare and community settings. Longitudinal evaluation is needed to determine whether post-training knowledge and intended application translate into sustained practice changes and improved HRSN and health outcomes.

## 1 Introduction

Population aging represents one of the most significant demographic shifts shaping health systems globally. In the United States, the population aged 65 years and older is projected to reach nearly 80 million by 2040, increasing the demand for coordinated healthcare and social services that support healthy aging (1). Older adults frequently experience multiple chronic conditions, complex medication regimens, functional limitations, and increased reliance on caregiving networks. These challenges are compounded by health-related social needs (HRSN), including food insecurity, transportation barriers, unstable housing, limited digital access and literacy, and social isolation (Table 1).

**Table 1:** Common health-related social needs (HRSN) identified among older adults.

Social need	Description	Potential CHW interventions
Food insecurity	Limited access to nutritious food or difficulty obtaining groceries	Referral to food assistance programs, community food banks, meal delivery services
Transportation barriers	Lack of transportation to healthcare appointments or services	Coordination of medical transportation and community ride services
Housing instability	Unsafe or unstable housing conditions affecting health	Connection to housing assistance programs and community support services
Social isolation	Limited social interaction leading to mental health risks	Referral to community programs, senior centers, and peer support networks
Digital access challenges	Limited access to technology or digital literacy	Training in telehealth use, device navigation, and patient portals
Financial strain	Difficulty affording medications or healthcare costs	Assistance with benefits enrollment and financial support programs

Evidence increasingly demonstrates that social determinants of health play a critical role in shaping health outcomes and healthcare utilization among older adults (2). Individuals facing unmet social needs are more likely to experience poorer chronic disease management, increased hospitalizations, and reduced quality of life. Addressing these challenges requires workforce models that extend beyond traditional clinical care to incorporate community-based approaches capable of navigating social and health systems simultaneously.

Health literacy is a critical component of effective care delivery for older adults. Health literacy encompasses both individual and organizational dimensions. Individual health literacy is the degree to which individuals have the ability to find, understand, and use information and services to inform health-related decisions and actions for themselves and others (3). Organizational health literacy is defined as the degree to which organizations enable individuals to find, understand, and use information and services to inform health-related decisions and actions for themselves and others (3). Health literacy influences medication adherence, disease management, and the ability to navigate complex healthcare systems (4). Older adults are disproportionately affected by limited health literacy due to cognitive changes, complex treatment regimens, and fragmented care environments, and multiple interacting health and social needs.

In this manuscript, health literacy is conceptualized as a cross-cutting HRSN-related need and barrier because it influences an individual’s ability to identify, understand, communicate about, navigate, and act on other health-related social needs. For example, a person may have access to transportation, food, medications, or community resources but still experience an unmet need if they cannot understand eligibility requirements, communicate their needs, locate appropriate services, or use health and social care information effectively. Organizational health literacy similarly influences whether systems make services and information accessible and actionable. Thus, health literacy provides a mechanism through which workforce capacity may influence the identification and navigation of multiple HRSNs.

Integrating health literacy strategies into workforce training, system strategy and navigation is therefore essential for improving patient engagement and health outcomes. Community health workers (CHWs) have emerged as an important component of community-based healthcare delivery. CHWs are trusted frontline public health workers who share lived experiences or cultural connections with the communities they serve (5). Their role includes health education, care navigation, resource coordination, and advocacy (5). Evidence shows that CHW interventions can improve chronic disease outcomes, increase preventive service utilization, and reduce healthcare costs (6, 7).

Despite growing recognition of the CHW workforce, limited attention has been paid to preparing CHWs specifically to address the complex needs of aging populations. Many existing CHW training programs focus primarily on maternal and child health, chronic disease prevention, or community outreach rather than geriatric care and aging systems navigation. As a result, there remains a gap in workforce training models that integrate health literacy, gerontology, and social care navigation.

To address this gap, the Health Literacy Lab, Community Health Worker Training Center, and Center for Older Adults at the University of North Texas Health Science Center developed a workforce innovation model designed to prepare CHWs and other frontline professionals to conduct health literacy-informed dementia and depression screening and facilitate referral for health-related social needs (HRSN) among older adults. The model integrates health literacy frameworks, community-engaged workforce development strategies, and cross-sector partnerships to strengthen workforce readiness for person-centered, age-friendly care across healthcare and community settings.

The purpose of this manuscript is to evaluate post-training knowledge, perceived skills, and intended application of evidence-based health literacy, dementia, and depression screening strategies among participants completing the training.

This leads to these aims:

1. Describe the development and implementation of a health literacy-centered CHW training model focused on aging populations.
2. Evaluate post-training knowledge, perceived skills, and intended application of evidence-based screening and communication strategies among participants.
3. Describe how the training model conceptualizes health literacy as a cross-cutting workforce competency relevant to HRSN screening, referral, and navigation among older adults.

By describing a health literacy-centered workforce training model and its post-training evaluation findings, this study contributes to efforts to strengthen workforce capacity for screening, communication, referral, and navigation among older adults with complex health and social needs. The findings provide preliminary evidence of post-training knowledge and reported readiness for application; they do not establish changes in practice or patient-level outcomes.

## 2 Materials and methods

### 2.1 Study design

This study used a descriptive post-training program evaluation to examine knowledge, perceived skills, and intended application of strategies among participants completing a community health worker (CHW) and frontline workforce training program.

The training model integrated health literacy principles into dementia and depression screening, communication, and referral strategies for older adults. The program was implemented through collaborative initiatives within the Health Literacy Lab, Community Health Worker Training Center, and Center for Older Adults at UNT Health in Fort Worth, Texas.

The evaluation examined participant knowledge using a post-training assessment and assessed participants’ perceptions of skills and intended application of selected health literacy-informed screening and referral strategies. Because the evaluation did not include a pre-training assessment, comparison group, or longitudinal follow-up, findings are interpreted as post-training outcomes and reported intentions rather than evidence of improvement or change over time.

The evaluation findings were used to inform a scalable workforce development model for preparing CHWs and frontline professionals to support health-related social needs (HRSN) screening and navigation among older adults.

### 2.2 Program setting and institutional context

The workforce training initiative was implemented through a collaborative academic, healthcare, community partnership model involving organizations across North Texas. Partner organizations included healthcare systems, community-based organizations serving older adults, social service providers, and public health agencies, creating a cross-sector foundation for workforce development focused on health literacy-informed care and health-related social needs (HRSN) screening and referral.

The Health Literacy Lab served as the central hub for curriculum development, research translation, and program evaluation. The Community Health Worker Training Center led workforce education and certification activities, while the Center for Older Adults contributed geriatric expertise, clinical perspectives, and community engagement. Together, these entities supported the development of an integrated training model designed to prepare CHWs and frontline professionals to better navigate the complex health and social needs experienced by older adults.

### 2.3 Curriculum development

Curriculum development followed a community-engaged framework that incorporated input from healthcare professionals, CHWs, older adults, caregivers, and community organizations. The curriculum integrated core competencies (Table 2) in eight domains.

**Table 2:** Core competencies for community health workers supporting older adults.

Competency domain	Key skills	Application in aging systems
Health literacy communication	Plain language, teach-back method, culturally responsive communication	Improve patient understanding of diagnoses, medications, and care plans
Chronic disease education	Education on diabetes, hypertension, cardiovascular disease, and dementia	Support self-management and preventive care
Medication literacy	Polypharmacy awareness, medication reconciliation, adherence support	Assist older adults managing complex medication regimens
Aging systems navigation	Medicare literacy, long-term care options, aging services	Guide patients through fragmented health and social systems
Social needs screening	Identification of food insecurity, housing instability, transportation barriers	Early detection of social determinants affecting health outcomes
Resource navigation	Referral to community resources and social services	Connect older adults to food programs, transportation, and housing assistance
Caregiver support	Education and resource coordination for family caregivers	Strengthen caregiver capacity and reduce caregiver burden
Digital health literacy	Support for telehealth, patient portals, and online resources	Reduce digital divide among older adults

Training modules emphasized practical skill development designed to prepare participants to address the complex health, social, and communication needs of older adults. Content included foundations of dementia and depression, evidence-based screening approaches, and health literacy-informed communication strategies to support appropriate identification and referral. Participants were trained in the use of screening tools and strategies for connecting older adults with appropriate health and community resources.

Participants also received training in caregiver support, recognizing the critical role family members and informal caregivers play in maintaining health, independence, and quality of life among older adults. Additional modules focused on screening for health-related social needs (HRSN) and identifying barriers that may affect an individual’s ability to access, understand, and benefit from healthcare services. To strengthen participants’ ability to support older adults navigating complex systems, training addressed community resource navigation, referral processes, and care coordination across healthcare and social service environments.

Given the increasing integration of technology into healthcare delivery, digital health literacy was incorporated to build participant skills related to accessing patient portals, telehealth services, and reliable online health information. Culturally responsive communication strategies were integrated throughout the curriculum to enhance participants’ ability to communicate effectively with individuals from diverse backgrounds, promote trust, and support equitable, person-centered care.

Training was delivered operationalizing conference presentations, in-person workshops, virtual sessions, and an on-demand self-learning module, with each training cohort or individual receiving approximately 1 to 2 hours of instruction in one session depending on the setting and time allotted. Instructional methods included formal lecture, interactive workshops or curriculum, case-based learning, role-playing exercises, and community-based practicum experiences depending on the delivery setting.

**Figure 1:**
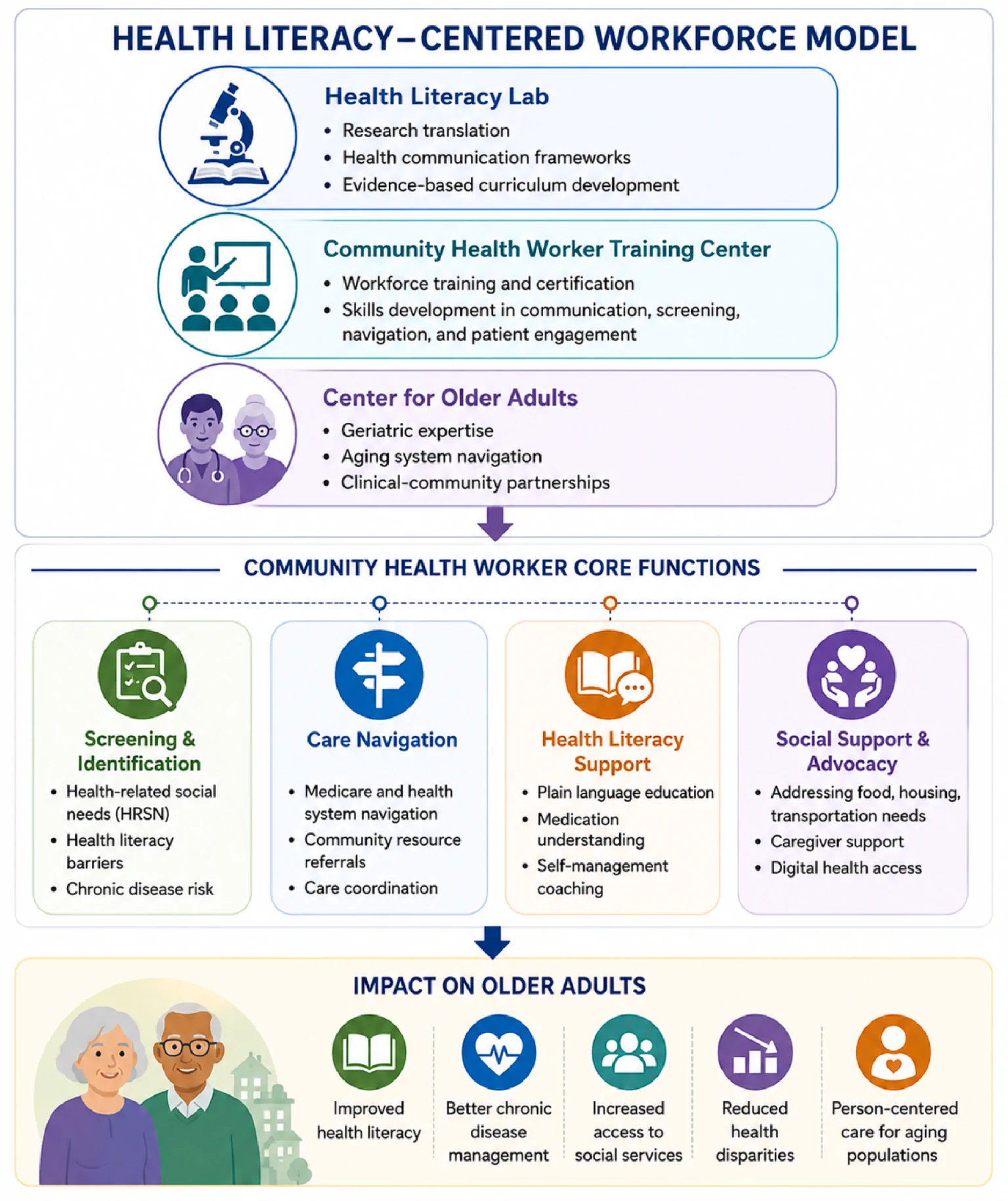
Health literacy–centered community health worker training model. Created using ChatGPT.

Together, these components informed the Health Literacy-Centered Community Health Worker Training Model (Figure 1), a workforce development framework designed to prepare CHWs and frontline professionals to support health literacy-informed screening, communication, referral, and navigation for older adults with complex health and social needs.

The Health Literacy–Centered Workforce Model offers a comprehensive framework for examining how investments in workforce development can influence health outcomes among older adults. The model posits that evidence-based health literacy practices, combined with specialized training in community health work and geriatric care, create a workforce equipped to bridge gaps between healthcare systems and community resources. Through core functions such as screening for health-related social needs, facilitating care navigation, delivering plain-language education, and providing social support, community health workers serve as critical intermediaries between older adults and the services they need.

The model therefore provides a conceptual basis for examining how workforce capacity building may contribute to health literacy, healthcare access, chronic disease management, and health equity among vulnerable aging populations. Importantly, these downstream outcomes were not measured in the current evaluation.

### 2.4 Workforce training participants

**Figure 2:**
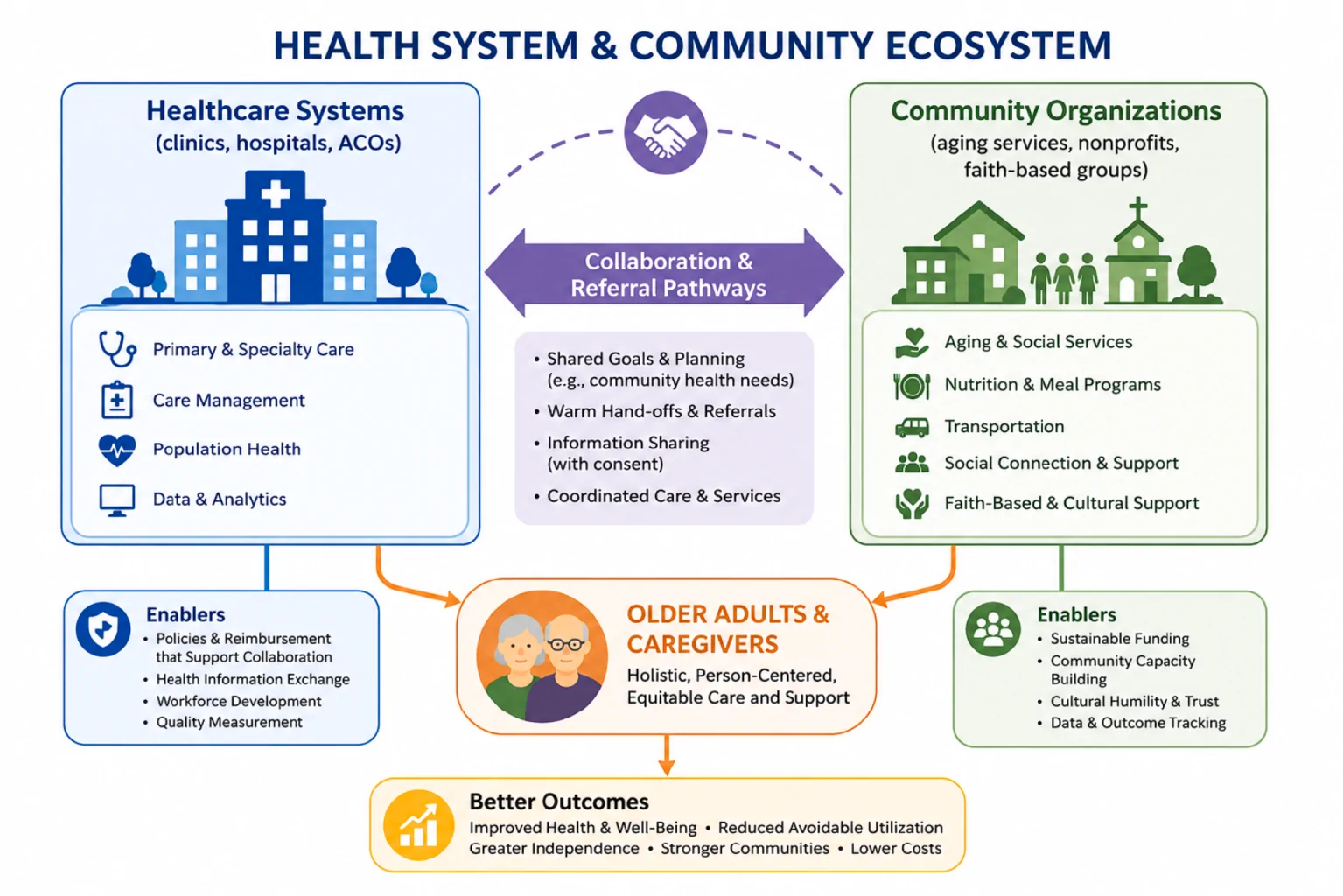
Health system and community ecosystem conceptual framework for aging systems. Created using ChatGPT.

Participants included community health workers, patient navigators, and frontline professionals, including paramedics cross-trained as CHWs, working across healthcare and community-based settings. Training cohorts were recruited through partnerships with healthcare systems, community organizations, and public health agencies guided by the Health System and Community Ecosystem conceptual framework (Figure 2), which informed cross-sector collaboration and coordinated workforce engagement.

Recruitment occurred through a combination of approaches including word-of-mouth. Invitation or application to present at conferences and regular continuing education (CE) meetings, and on demand offering of the self-learning module. Participants were eligible if they were CHWs or front-line trusted messengers seeking additional training in older adult care or attending various conferences or meetings when featured. Participation in the post-training evaluation was included in the presentation time and at the end of the self-learning module in order to receive the continuing education certificate. Participants represented diverse professional backgrounds and served urban and rural communities experiencing health inequities and increased social vulnerability.

The Health System and Community Ecosystem model situates CHWs work within a broader framework of clinical-community integration, recognizing that health outcomes among older adults are influenced by both healthcare delivery systems and community-based supports. The model underscores the role of collaborative partnerships, bidirectional referral pathways, and coordinated service delivery in addressing social determinants of health, health literacy barriers, and unmet social needs. As such, it provides a conceptual foundation for understanding how workforce development and cross-sector collaboration can enhance access to care, improve chronic disease management, and reduce inequities among vulnerable aging populations.

### 2.5 Evaluation measures

#### 2.5.1 Participant characteristics

A total of 270 participants completed the training, representing a diverse interdisciplinary workforce that included community health workers (CHWs), nurses, social workers, physicians, pharmacists, educators, students, and other health professionals. Knowledge was assessed using a post-training evaluation completed by 201 participants. Thus, 201 of the 270 participants who completed the training completed the post-training assessment, representing a 74.4% response rate. This might have been impacted by whether the specific discipline needed the certificate for CE reporting.

The post-training assessment was administered immediately following training to be completed within the training timeframe and took approximately 5 min. The assessment included measures of post-training knowledge and participant-reported perceptions of skills and intended application of selected training strategies.

#### 2.5.2 Post-training knowledge assessment

The mean post-training knowledge score among participants was 84.9% (SD = 9.23), This finding indicates a high level of performance on the post-training knowledge assessment but cannot be interpreted as evidence of knowledge gain because pre-training knowledge was not measured. Physicians (*n* = 6) achieved the highest average post-training score (90.0%), closely followed by CHWs (89.0%; *n* = 32), while students (85.6%), social workers (85.9%), nurses (84.3%), and healthcare administrators (84.0%) also demonstrated strong performance. Average scores were not reported for professions with insufficient responses. However, there were sufficient responses from 16 different disciplines.

#### 2.5.3 Knowledge assessment findings

Participants demonstrated strong knowledge of dementia, depression, health literacy, and culturally responsive communication following completion of the training (Table 3). Nearly all participants recognized the importance of plain language communication, the influence of health literacy and language barriers on screening, and the relationship between depression and dementia. Participants also demonstrated strong understanding of appropriate referral practices and recognition of common dementia symptoms. Knowledge related to the purpose of cognitive screening and identification of the Montreal Cognitive Assessment (MoCA) as a dementia screening tool was comparatively lower, suggesting opportunities for reinforcing these concepts in future trainings.

**Table 3:** Post-training knowledge assessment results (*N* = 201).

Knowledge item	Correct *n* (%)
Depression in older adults can worsen dementia symptoms	198 (98.5%)
Provide written guidance in plain language to communicate effectively	198 (98.5%)
Health literacy and language barriers affect whether individuals receive screening	200 (99.5%)
Refer patients for a comprehensive mental health evaluation following a positive depression screen	186 (92.5%)
Cultural differences influence how dementia and depression symptoms are expressed and understood	188 (93.5%)
PHQ-9 is an appropriate screening tool for depression	177 (88.1%)
Persistent short-term memory loss is a common symptom of dementia	171 (85.1%)
Health literacy helps professionals communicate screening results effectively	165 (82.1%)
Primary purpose of cognitive screening is early identification and timely referral, not diagnosis	102 (50.7%)
Montreal Cognitive Assessment (MoCA) is an appropriate dementia screening tool	124 (61.7%)

#### 2.5.4 Training impact and anticipated practice change

The findings can be descriptively mapped to the first three levels of the Kirkpatrick Model of Training Evaluation: Reaction, Learning, and intended Behavior. Because the evaluation was post-training only, the Behavior level should be interpreted as reported intention rather than observed behavior change, and the Results level was not assessed.

*Level 1: Reaction*. Participants viewed the training as valuable and applicable to their professional roles. Respondents anticipated that the training would improve patient engagement, patient health outcomes, and patient safety, indicating that the content was perceived as relevant to the care of older adults. Participants also identified potential implementation barriers, including limited opportunities to encounter appropriate patient situations, organizational and system-level constraints, reimbursement and billing considerations, and time limitations. These findings suggest that participants were engaged with the training and were able to critically consider how the strategies might be integrated into real-world practice settings.

*Level 2: Learning*. Evidence of learning was demonstrated through the post-training knowledge assessment, in which participants achieved a mean score of 84.9% (SD = 9.23). These results indicate that participants acquired knowledge related to evidence-based dementia and depression screening, health literacy-informed communication, and referral practices for older adults. Because no pre-training assessment was conducted, the findings cannot establish the magnitude of knowledge acquisition attributable to the training.

*Level 3: Behavior*. The evaluation also demonstrated participants’ intention to change practice behaviors. Among respondents, 167 participants (83.1%) reported that they planned to implement the screening and communication strategies learned during the training when working with older adults. Participants represented a diverse range of practice settings, including primary care (*n* = 38), medically underserved communities (*n* = 39), and rural settings (*n* = 27). These findings demonstrate reported intention to apply the strategies across multiple healthcare and community environments; however, actual implementation was not measured.

*Level 4: Results*. The current evaluation did not assess Level 4 outcomes, such as changes in screening rates, referral patterns, healthcare utilization, or patient health outcomes. Future evaluation efforts should examine whether implementation of the training leads to increased dementia and depression screening among older adults, improved referral and follow-up processes, enhanced patient engagement and medication understanding, and measurable improvements in patient safety and health outcomes. Assessing these outcomes would provide important evidence regarding the broader impact of the training on healthcare delivery and older adult well-being.

Overall, applying the Kirkpatrick framework demonstrates that the training was well received (Level 1), resulted in meaningful knowledge acquisition (Level 2), and showed strong potential for practice change (Level 3), while highlighting the need for future research to evaluate longer-term organizational and patient-level outcomes (Level 4).

### 2.6 Ethical considerations

This study was conducted in accordance with institutional guidelines for research involving human participants. Evaluation activities involving program participants were reviewed by the North Texas Regional Institutional Review Board (IRB) at UNT Health Fort Worth to ensure compliance with ethical standards.

## 3 Results

### 3.1 Workforce training implementation

The workforce development program successfully implemented multiple training cohorts involving community health workers and frontline professionals serving older adults. Training sessions emphasized interdisciplinary collaboration and practical strategies for addressing social determinants of health.

Participants reported post-training understanding of aging systems, screening tools, and ability to refer clients to or assist them in service utilization, including community-based services, and long-term care resources.

### 3.2 Post-training health literacy competencies

Post-training assessments demonstrated that participants reported confidence in applying health literacy-informed communication and evidence-based screening strategies when working with older adults. Participants indicated readiness to apply skills in:

- Using plain language communication
- Assessing patient understanding
- Screening for dementia and depression
- Facilitating appropriate referral to services for identified needs

Together with the strong post-training knowledge assessment scores and participants’ intention to incorporate these strategies into practice, findings indicate that participants reported competencies relevant to future HRSN screening and referral activities involving older adults. This provides preliminary evidence of post-training workforce readiness but does not establish improvement in competency or actual practice change. These competencies are particularly relevant for supporting individuals with multiple chronic conditions, complex treatment plans, and health-related social needs.

### 3.3 Health literacy as a cross-cutting component of HRSN screening and navigation

This study provides preliminary support for the use of a health literacy-centered training approach in future HRSN screening and referral education for community health workers serving older adults.

Participants demonstrated strong post-training knowledge and reported confidence and intention to apply evidence-based screening and communication strategies. The interdisciplinary composition of the training cohorts also illustrates the potential feasibility of engaging healthcare and community-based professionals within a shared workforce development model.

Health literacy is particularly relevant to HRSN because the ability to identify and respond to social needs depends not only on whether services exist but also on whether individuals can find, understand, communicate about, and use information related to those services. In this conceptualization, health literacy functions as a cross-cutting competency that may influence navigation of other HRSNs, including transportation, food access, medication-related needs, social support, and digital access.

Although these HRSNs were incorporated into the training framework, the current evaluation did not directly measure participants’ ability to identify or resolve individual HRSNs such as food insecurity, transportation barriers, housing instability, or medication-related needs. Rather, the findings demonstrate post-training knowledge and reported readiness related primarily to dementia, depression, health literacy, communication, screening, and referral.

These findings support the premise that health literacy can serve as a cross-cutting workforce competency for HRSN screening and navigation and provide a rationale for future evaluations that directly measure HRSN identification, referral, and resolution.

## 4 Discussion

### 4.1 Advancing workforce innovation in aging systems

The findings highlight the importance of workforce development in preparing community health workers and other frontline professionals to address the complex health and social needs of aging populations. Traditional healthcare models often provide limited training in health literacy, age-friendly care, and HRSN screening and referral, creating opportunities to strengthen workforce capacity through targeted education.

The current evaluation demonstrated strong performance on a post-training knowledge assessment and high reported intention to apply selected screening and communication strategies. These findings suggest that the training model is feasible to implement across a diverse interdisciplinary workforce and provides a foundation for future evaluation of workforce application and organizational outcomes.

Participants demonstrated strong post-training knowledge, reported confidence in selected competencies, and high intention to apply the strategies addressed during training. These findings provide preliminary evidence of workforce readiness but do not establish whether training resulted in sustained changes in practice.

Future implementation research should evaluate whether these improvements translate into sustained changes in screening practices, referrals, and patient outcomes.

### 4.2 The role of health literacy in addressing HRSN

Health literacy is a foundational component of effective care for older adults and represents an important cross-cutting mechanism linking healthcare access, communication, screening, and navigation. In the context of HRSN, health literacy influences whether individuals can recognize a need, understand available resources, communicate their circumstances, determine eligibility, navigate systems, and act on recommendations.

This relationship is particularly important among older adults who may experience multiple simultaneous needs. For example, transportation barriers may require an individual to understand eligibility criteria and scheduling processes; food insecurity may require navigating community resources and benefit programs; medication-related challenges may require understanding instructions and communicating questions; and limited digital access may be compounded by difficulty understanding online health information or using patient portals.

Accordingly, this manuscript conceptualizes health literacy as a cross-cutting HRSN-related need rather than as a discrete social need operating independently of other HRSNs. The distinction is important because the current evaluation did not directly measure the prevalence or resolution of individual HRSNs. Instead, it evaluated workforce competencies that may enable CHWs to identify, communicate about, and navigate those needs.

The Health Literacy-Centered Community Health Worker Workforce Model (Figure 2) integrates research translation, workforce development, geriatric expertise, health literacy-informed communication, HRSN screening, and community resource navigation. The post-training findings provide preliminary support for the feasibility of teaching these interconnected competencies to a diverse workforce.

Importantly, the model proposes a pathway rather than demonstrating a causal relationship. Workforce training may strengthen knowledge and readiness; health literacy-informed practice may then facilitate communication, screening, and navigation; and these activities may ultimately contribute to improved access and health outcomes. The latter stages of this pathway require prospective evaluation.

### 4.3 Implications for health equity

Older adults experiencing social vulnerability face significant barriers to accessing healthcare and social services. CHWs are uniquely positioned to address these disparities due to their community connections, cultural responsiveness, and ability to bridge healthcare and community systems.

The findings suggest that a health literacy-centered workforce approach can provide a common foundation for interdisciplinary professionals working with older adults. By emphasizing plain-language communication, assessment of understanding, screening, referral, and navigation, the model addresses workforce competencies that are relevant across healthcare and community settings. Thus, demonstrating health literacy-centered workforce training can strengthen community-based capacity to promote health equity.

The health equity relevance of this approach lies not in demonstrating that the training reduced disparities, which was not evaluated, but in strengthening workforce capacity to recognize and navigate communication and access barriers that may contribute to inequitable use of health and social services.

### 4.4 Limitations

Several limitations should be acknowledged.

First, this was a post-training evaluation without a pre-training assessment or comparison group. Therefore, the study cannot determine whether participants’ knowledge, skills, or confidence improved because of the training.

Second, 201 of the 270 participants who completed the training completed the post-training assessment, representing a 74.4% response rate. Nonresponse may have introduced response bias if participants who completed the assessment differed systematically from those who did not.

Third, several outcomes were self-reported, including perceived skills and intended application. These measures may be influenced by social desirability and recall bias and should not be interpreted as evidence of observed practice change.

Fourth, the evaluation primarily measured knowledge and reported readiness related to dementia, depression, health literacy, communication, screening, and referral. Although HRSNs were incorporated into the curriculum and conceptual framework, the evaluation did not directly measure changes in HRSN identification, referral, resolution, healthcare utilization, or patient outcomes.

Fifth, the evaluation was conducted within a specific regional and partnership context, which may limit transferability to other settings.

Finally, the current evaluation did not assess longitudinal outcomes or Kirkpatrick Level 4 results, including changes in organizational practices, screening rates, referral completion, healthcare utilization, or older adult health outcomes.

### 4.5 Future directions

Future research should incorporate longitudinal and multisite evaluation approaches to determine whether post-training knowledge and reported intentions translate into observed workforce behaviors and organizational outcomes.

Future research should explore:

- Pre/post evaluation designs to assess changes in knowledge, confidence, and skills attributable to training.
- Longitudinal evaluation of CHW-led interventions to examine changes in HRSN screening, referral practices, care coordination, and older adult outcomes.
- Direct measurement of HRSNs, including food insecurity, transportation barriers, social isolation, medication-related needs, and digital access.
- Evaluation of health literacy as a cross-cutting mechanism influencing the identification, navigation, and resolution of other HRSNs.
- Assessment of referral completion and connection to community resources.
- Integration of CHWs into value-based care models and healthcare delivery systems that emphasize prevention, care coordination, and addressing social drivers of health; and
- Development of workforce strategies supporting geriatric CHW specialization and standardized health literacy-centered training approaches.

Future studies should therefore test the conceptual pathway proposed by the Health Literacy-Centered Workforce Model: workforce training → health literacy-informed knowledge and skills → observed screening, communication, referral, and navigation behaviors → connection to needed services → patient- and system-level outcomes.

This sequence would allow future research to determine whether the competencies evaluated in the current study translate into measurable improvements in HRSN identification and navigation and, ultimately, health outcomes.

## 5 Conclusion

Addressing the health-related social needs of aging populations requires workforce capacity to bridge healthcare and community-based systems. This study describes a Health Literacy–Centered Community Health Worker Training Model and the Health System and Community Ecosystem Model that integrate health literacy, geriatric care, screening, communication, referral, and community resource navigation into workforce development. Among 270 participants who completed training, 201 completed the post-training assessment. Participants demonstrated strong post-training knowledge and reported confidence and intention to apply selected screening and communication strategies.

These findings provide preliminary evidence supporting the feasibility of a health literacy-centered approach to workforce development and support the conceptualization of health literacy as a cross-cutting HRSN-related competency. Health literacy may influence an individual’s ability to identify, understand, navigate, and act on other health-related social needs, making it particularly relevant to CHW screening, referral, and navigation activities.

However, the current post-training evaluation did not establish changes in practice, HRSN identification or resolution, healthcare utilization, or older adult health outcomes. Future longitudinal and multisite studies should determine whether the knowledge and intended application observed after training translate into sustained workforce behaviors, improved HRSN screening and referral, stronger clinical-community coordination, and measurable improvements in outcomes for older adults.

## Data Availability

The raw data supporting the conclusions of this article will be made available by the authors, without undue reservation.
